# Generation and Stability of Size-Adjustable Bulk Nanobubbles Based on Periodic Pressure Change

**DOI:** 10.1038/s41598-018-38066-5

**Published:** 2019-02-04

**Authors:** Qiaozhi Wang, Hui Zhao, Na Qi, Yan Qin, Xuejie Zhang, Ying Li

**Affiliations:** Key Laboratory of Colloid and Interface Chemistry of State Education Ministry, Shandong University, Jinan, 250100 P. R. China

## Abstract

Recently, bulk nanobubbles have attracted intensive attention due to the unique physicochemical properties and important potential applications in various fields. In this study, periodic pressure change was introduced to generate bulk nanobubbles. N_2_ nanobubbles with bimodal distribution and excellent stabilization were fabricated in nitrogen-saturated water solution. O_2_ and CO_2_ nanobubbles have also been created using this method and both have good stability. The influence of the action time of periodic pressure change on the generated N_2_ nanobubbles size was studied. It was interestingly found that, the size of the formed nanobubbles decreases with the increase of action time under constant frequency, which could be explained by the difference in the shrinkage and growth rate under different pressure conditions, thereby size-adjustable nanobubbles can be formed by regulating operating time. This study might provide valuable methodology for further investigations about properties and performances of bulk nanobubbles.

## Introduction

Nanobubbles are gaseous domains which could be found at the solid/liquid interface or in solution, known as surface nanobubbles (SNBs)^1,2^ and bulk nanobubbles (BNBs)^3^, respectively. For BNBs, generally recognized as spherical bubbles with the diameter of less than 1μm surrounded by liquid, though it has been observed firstly in 1981^4^, the existence of long-lived BNBs is still a controversial subject as it is contrary to the classical theory^5,6^. Recently, BNBs have been gradually revealed that it has not only unique physiochemical properties, but also great application prospect in many fields, such as biomedical imaging and targeted treatment^7,8^, hydrogen storage^9^, water treatment^10,11^, cleaning^12^ and promoting the metabolism of living organisms^13,14^, which makes the investigation on BNBs have captured increasing interest.

In the past decade, diverse methods have been used to produce bulk nanobubbles including cavitation, electrolysis, ultrasonication coupled with Pd-coated electrodes, temperature gradients, and so on^15–20^, in which cavitation and electrolysis were used more often. Cavitation, mainly referring to hydrodynamic cavitation, usually occurs in flowing fluid where the local pressure is lower than the critical value. A dispersion of cavitation nanobubbles is collected according to the difference in the rising velocity described by Hadamard-Rybczynski equation^21,22^. Microbubbles would disperse relatively rapidly up to gas-liquid surface and burst, while nanobubbles would remain in solution owing to slow rise. As a result, it usually takes time to obtain nanobubbles and is difficult to accurately control the size of cavitation bubbles. The size-controllable BNBs can be achieved by regulating the applied voltage in electrolyzing water^23^, but there are some limitations in the types of generated nanobubbles (usually O_2_ and H_2_). In some of potential applications of BNBs, such as gas transportation in the hypoxic tumor and biomedical imaging^24–26^, rational size range and meaningful gas kinds of formed BNBs are both required to improve the performance of bubbles. It would be of significance to explore novel formation method of size-adjustable BNBs with different gases.

In this paper, the adjustable N_2_, O_2_, and CO_2_ BNBs were generated by using periodic pressure change method. The existence of nanobubbles was testified by Tyndall effect and the images of freeze-fracture transmission electron microscope (FF-TEM), and the size distribution was investigated using light scattering method. The stability of generated BNBs was also investigated by the time-dependent size change. In addition, the generation mechanism of BNBs was discussed and verified by the dependence of size on periodic pressure change time.

## Methods

### Materials

In our study, ultrapure water with a resistivity of 18.2 MΩ was used to generated nanobubbles, which was obtained using a ULPYT-31110 water purification system (Youpu Ultrapure Technology Co., Ltd., P. R. China), having ultralow heavy metal content (≤0.1 ppb) and the number of microparticles (≤1/ml). In order to avoid the interference of potential contamination and microorganism, the ultrapure water was filtered through 0.1μm pore size filter before it was used to prepare all solution. The vessels were cleaned with detergent, 10% NaOH solution and rinsed with plenty of tap water and ultrapure water before use. Prior to use, the pH of aqueous solution was adjusted to the demand value by adding HCl (AR grade) and NaOH (AR grade). N_2_, O_2_ and CO_2_ (purity > 99.999%) were purchased from Deyang Special Gas Co., Ltd.

### Device and operational details of generated BNBs

**Figure 1:**
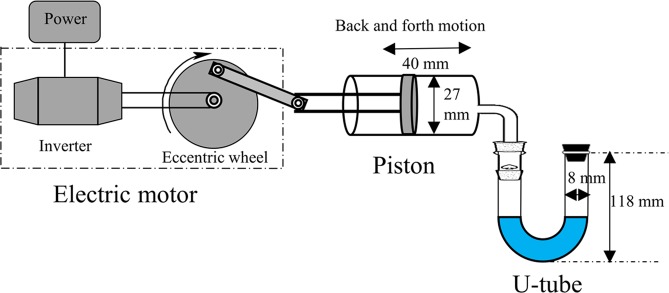
The schematic diagram of generating bulk nanobubbles.

BNBs were generated by using the periodic pressure change device designed by ourselves, as shown schematically in Fig. 1, which mainly consists of electric motor, piston and transparent U-tube. The work principle is that, the linear motion of piston back and forth converted by rotation of the eccentric wheel under motor driving, causes the periodic change of internal pressure in U-tube. Consequently, the gas solubility in aqueous solution would change, which brings about the production of nanobubbles.

In a typical process, the device operated three times firstly in the room, each time for 10 min ahead of normal work to further exclude the potential impurities. After that, the device was filled with gas, and 3 ml solution pre-saturated with this gas by bubbling for 30 min was quickly poured into U-tube prior to BNBs generation. Then the inlet (the position of stopper at end of the U-tube in Fig. 1) was sealed immediately to prevent the gas from escaping. During work process, some macro-bubbles can be observed on the U-tube wall. After generation, the solution was sealed in vial with parafilm and stored at low temperature. Unless otherwise stated, the motor frequency and working time were first set to 60 r/min and 120 min in our experiment.

### Particle size measurement

The size of nanobubbles was measured by dynamic light scattering (DLS, Zetasizer Nano ZS, Malvern), which was performed at a scattering angle of 173 at the temperature of 25 °C. The refractive index of material was set to 1 in accord with air^27^. In measurement, the velocity of particles that are doing the Brownian motion is detected by monitoring the undulation of scattered light, and then converts to particle size by the Stokes-Einstein equation, so the original signal obtained from the instrument is the scattered light of sample particles. Because the intensity of scattering light following the Rayleigh scattering law is proportional to the sixth power of particle diameter, the signal of larger particles is enlarged than that of the smaller ones. In this paper, the intensity and number distribution were both presented in order to demonstrate the population of nanobubbles in solution clearly.

### Zeta potential measurement

The zeta potential of BNBs was measured by laser doppler velocimetry (LDV) (Zetasizer Nano ZS, Malvern). A curved capillary sample cell (DTS1070) was used. Automatic runs (1–100) for each sample was performed, and the time interval between each measurement was 30 s. To investigate the stability of generated BNBs, the variation of size distribution and zeta potential with time were both determined at interval of 24 h. All measurements were carried out at least three times at 25 °C.

### Freeze-fracture transmission electron microscope

The FF-TEM usually used to study biological microstructures offers an effective way for observing the morphology of bulk nanobubbles^28,29^. The details in the freeze-fracture replica film preparation have been described in reference^30^, so it was briefly described here. A small amount of sample (~4 μl) was taken out from vial and mounted onto a specimen holder, which was immersed in liquid nitrogen later at once. For fracturing and replication, a freeze-fracture apparatus (EM BAF 060, Leica, Germany) was used at a temperature of −170 °C. Pt/C was deposited at a 45° angle to shadow the replicas, and C was deposited at a 90 angle to consolidate the replicas. The replicas were transferred onto a copper grid and then examined with a JOEL JEM-1011(100 kV) transmission electron microscope (TEM).

## Results and Discussion

### Generation of bulk nanobubbles

**Figure 2:**
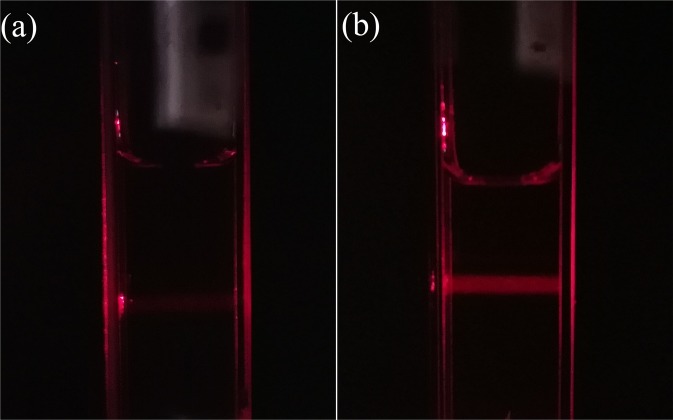
The scattering of a laser beam passing through the cuvette filled with nitrogen-saturated ultrapure water (**a**) before and (**b**) after the periodic pressure change for 120 min.

The Tyndall effect based on light scattering is a direct and easy method to reflect the microcosmic variation of solution. The variation of nitrogen-saturated ultrapure water solution was observed using the laser beam in darkroom, shown in Fig. 2. Compared with nitrogen-saturated ultrapure water in Fig. 2(a), a bright path in the vertical direction of incident light can be clearly seen in nitrogen-saturated ultrapure water solution with employing the periodic pressure change (Fig. 2(b)), which demonstrates there are colloid particles in solution after the periodic pressure change. Because the introduction of impurities is not likely to occur during experiment, the colloid particles most likely be nitrogen nanobubble population.

**Figure 3:**
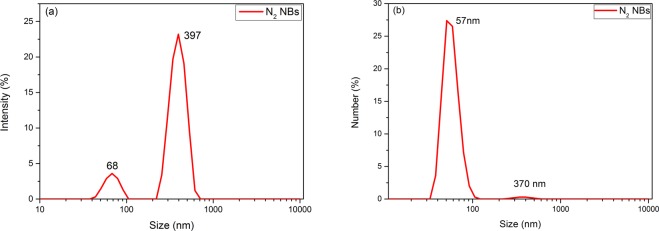
The size distribution of nitrogen nanobubbles in aqueous solution determined immediately after employing the periodic pressure change for 120 min. (**a**) The intensity distribution and (**b**) the number distribution.

The size of nitrogen nanobubbles in aqueous solution was determined immediately by DLS after employing the periodic pressure change, as depicted in Fig. 3. There was a regular bimodal curve with peak values around 68 nm and 397 nm respectively. By knowable principles, large number of the nitrogen BNBs generated by employing periodic pressure change have a hydrodynamic diameter less than 100 nm, of which the most probable diameter is about 57 nm, and there are small number of the bubbles with a diameter of about several hundred nm (shown in Fig. 3(b)). In light scattering measurement, the scattering signal is ultrasensitive to impurities, as in the system of ethanol and water mixture studied by Häbich *et al*.^31^, it was found there is no obvious light scattering of bulk nanobubbles when the impurities (mainly ethanol, isopropanol and acetone) in AR grade ethanol are removed by distillation. Therefore, the DLS measurement of nitrogen-saturated ultrapure water without applying periodic pressure change was performed to exclude the possibility that the impurities in water made a big contribution to the scattering signal of DLS. As it was shown in Fig. S1 in the supplementary information, the wide size distribution, the micro-peak appearance and poor repeatability could be understood that the content of impurities in water was very low, even lower than the limit of minimum detection level of the instrument. It could be confirmed that the distinct DLS signal of nitrogen-saturated ultrapure water after applying periodic pressure change is contributed by the formed nanobubbles.

**Figure 4:**
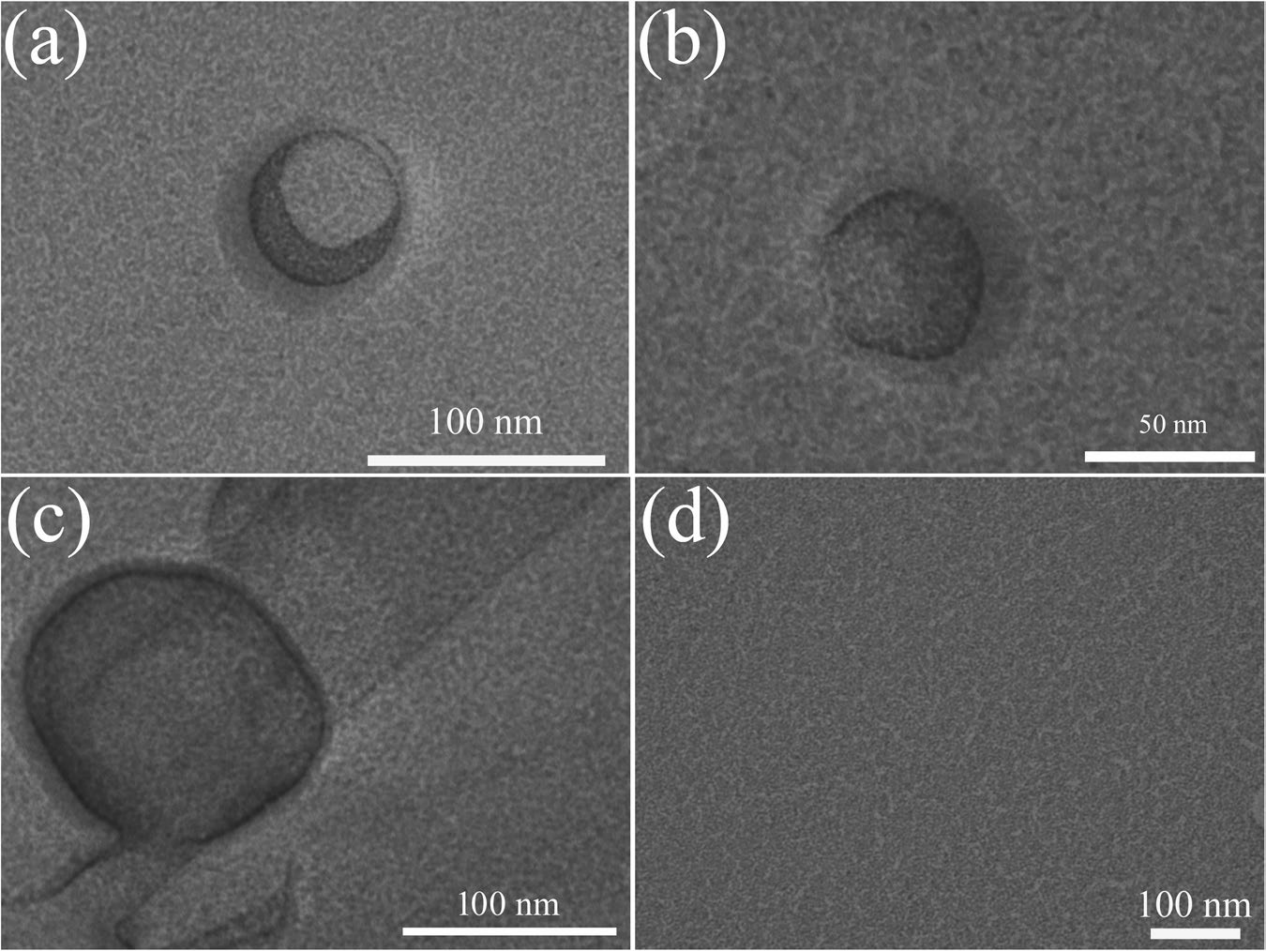
FF-TEM images of nitrogen nanobubbles formed in ultrapure water after employing the periodic pressure change for 120 min. (**a–c**) were nitrogen nanobubbles; (**d**) was the nitrogen-saturated ultrapure water.

Additionally, the images of N_2_ nanobubbles formed in ultrapure water after employing the periodic pressure change were observed by means of the FF-TEM. Because there are some nanobubbles which were not be well replicated on the replica film, as marked nanobubbles by red numbers in Fig. S2 (in the supplementary information), the TEM images containing one nanobubbles were shown in Fig. 4 to better show the morphology of formed nanobubbles. It demonstrated that the spherical holes with diameters about 60 nm and 200 nm on freeze-fractured cross-section can be vividly observed, which provide a direct evidence that the existence of the nanobubbles in ultrapure water after employing the periodic pressure change. Therefore, it can be confirmed that nitrogen BNBs with the mainly hydrodynamic diameter less than 100 nm, can be generated by employing periodic pressure change.

### Stability of generated nitrogen BNBs

**Figure 5:**
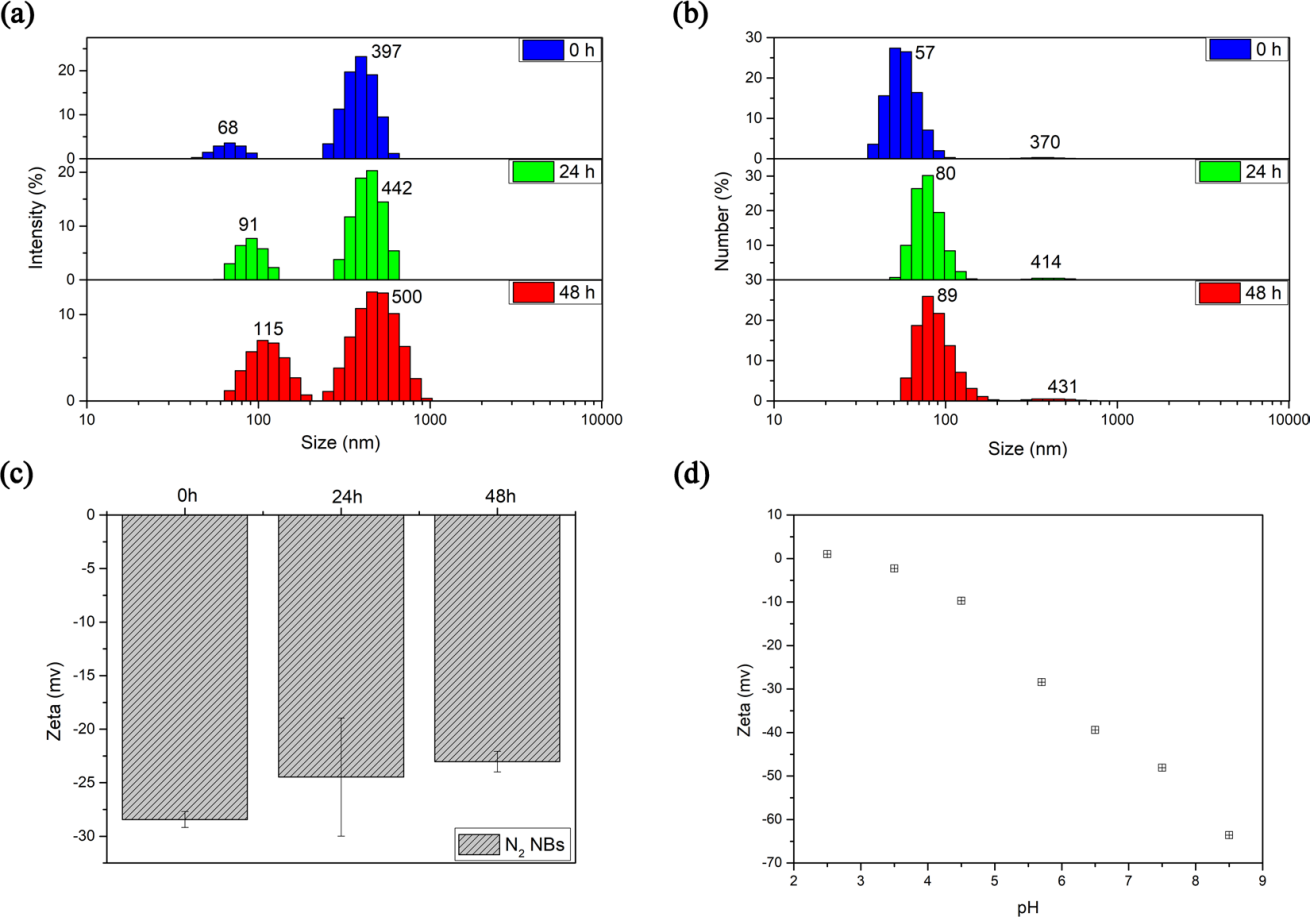
The size distributions and zeta potential of N_2_ nanobubbles formed in aqueous solution after employing the periodic pressure change for 120 min. (**a**) The intensity distribution with time and (**b**) the number distribution with time. (**c**) The zeta potential variation of N_2_ nanobubbles with time. (**d**) The zeta potential variation of N_2_ nanobubbles with different pH. Vertical bar indicates the standard deviation of three measurements.

The lifetime of the formed nitrogen nanobubbles was studied by investigating the change of bubble size with time. Figure 5(a) showed that N_2_ nanobubbles have excellent stability, which can remain in solution for more than 48 h. The hydrodynamic diameter of formed BNBs increased from the initial most probable diameters about 68 nm and 397 nm to 91 nm and 442 nm over 24 h respectively. After 48 h, the most probable diameters became 115 nm and 500 nm. Both sizes of nanobubbles became larger with the lapse of time, Ostwald ripening rule is likely to be followed during nanobubbles growth^32–34^. The number size distribution of the formed N_2_ BNBs in Fig. 5(b) agreed well with the growth tendency. The excellent stability of formed nitrogen nanobubbles population may suggest that once BNBs are formed, it will remain in solution for a long time.

As we all know, for small bubbles, it should be more unstable according to the Yong-Laplace equation shown in following eq. (1):

1 $${{\rm{P}}}_{{\rm{in}}}={{\rm{P}}}_{{\rm{o}}}+2{\rm{\sigma }}/{\rm{r}}$$

P_in_ is the internal pressure of bubble, P_o_ is the ambient pressure,σ is the surface tension of liquid and r is the radius of bubble. For example, the P_in_ is about 1.54 MPa when r is 100 nm (P_o_ is atmospheric pressure, σ is about 72 mN/m). BNBs would dissolve instantly under this overpressure because it is more difficult to sustain the gas equilibrium between surrounding solution and nanobubbles. As the theoretical prediction, the lifespan of BNBs with diameter smaller than 1000 nm is less than 0.02s^35^.

However, BNBs possessing unexpected stability in experiment have been reported in literatures, which is inconsistent with the above prediction. The generated nanobubbles by using a baffled high intensity agitation (BHIA) cell could exist for 5 h in surfactants-free solution, and up to 24 h in presence of surfactants^36^. Also, Oh and Kim^34^ found that the CO_2_ nanobubbles fabricated by a gas-liquid mixing method has high concentration over 24 h (up to (2.94 ± 0.16) × 10^8^ particles/ml with the mean diameter increasing to 110.00 ± 4.58 nm). Even O_2_ nanobubbles with the diameter of 137 nm remain to be detected by DLS on sixth day in absence of additives^27^.

The reason for unexpected stability is still unclear though it has been discussed for decades. Some investigations have suggested the negative electrical charge at the gas-liquid interface is a main factor for stabilizing BNBs, which prevents bubble coalescence by repelling each other^37,38^. Therefore, the zeta potential of formed N_2_ nanobubbles was determined using electrophoresis method. The zeta potential variation of N_2_ nanobubbles within 48 hours was demonstrated in Fig. 5(c). The N_2_ nanobubbles were negatively charged with average zeta potential about −28.43 ± 0.75 mv at pH 5.7, which is in line with the reported results in the literature^39^. The zeta potential value slightly increased to −24.47 ± 5.51 mv over 24 h, and it became −23.03 ± 0.96 mv when the standing time is up to 48 h.

The negative electrical charge at gas-liquid interface of BNBs could be explained by the preferential adsorption of hydroxyl ions to interface. It is interpreted that hydrogen ions are more inclined to remain in solution owing to the energy difference in hydrous enthalpy of OH^−^ (−446.8 kJ/mol) and H^+^ (−1104 kJ/mol)^19,40^, or the orientation of water dipole at the interface cause the attraction of OH^−^ to the interface^17,41,42^. In this paper, to confirm that the zeta potential of the formed nanobubbles in this study is associated with OH^−^, the influence of pH on zeta potential was determined. Figure 5(d) showed that the pH of the solutions do have a strong effect on the zeta potential of formed nanobubbles. The zeta potential values were negative within a wide pH range, and rapidly decreased when pH value was over 4.5, which can ascribe to the adsorption of OH^−^ to the interface. It also showed that the isoelectric point of N_2_ nanobubbles is between 2 and 3 as reported in literature^43^.

In summary, the zeta potential measurements showed that the zeta potential of N_2_ nanobubbles had a high absolute value, and the potential experienced no significant change with time. Therefore, the negative zeta potential has positive contribution for the stability of BNBs by preventing the coalescence between nanobubbles through electrostatic repulsion interaction, which could be noted as a contribution factor for the stability of BNBs, apart from forming supersolid skin and introducing diffusive shielding of BNBs clusters reported previously^44–46^.

### Generation and stability of O_2_ and CO_2_ nanobubbles

**Figure 6:**
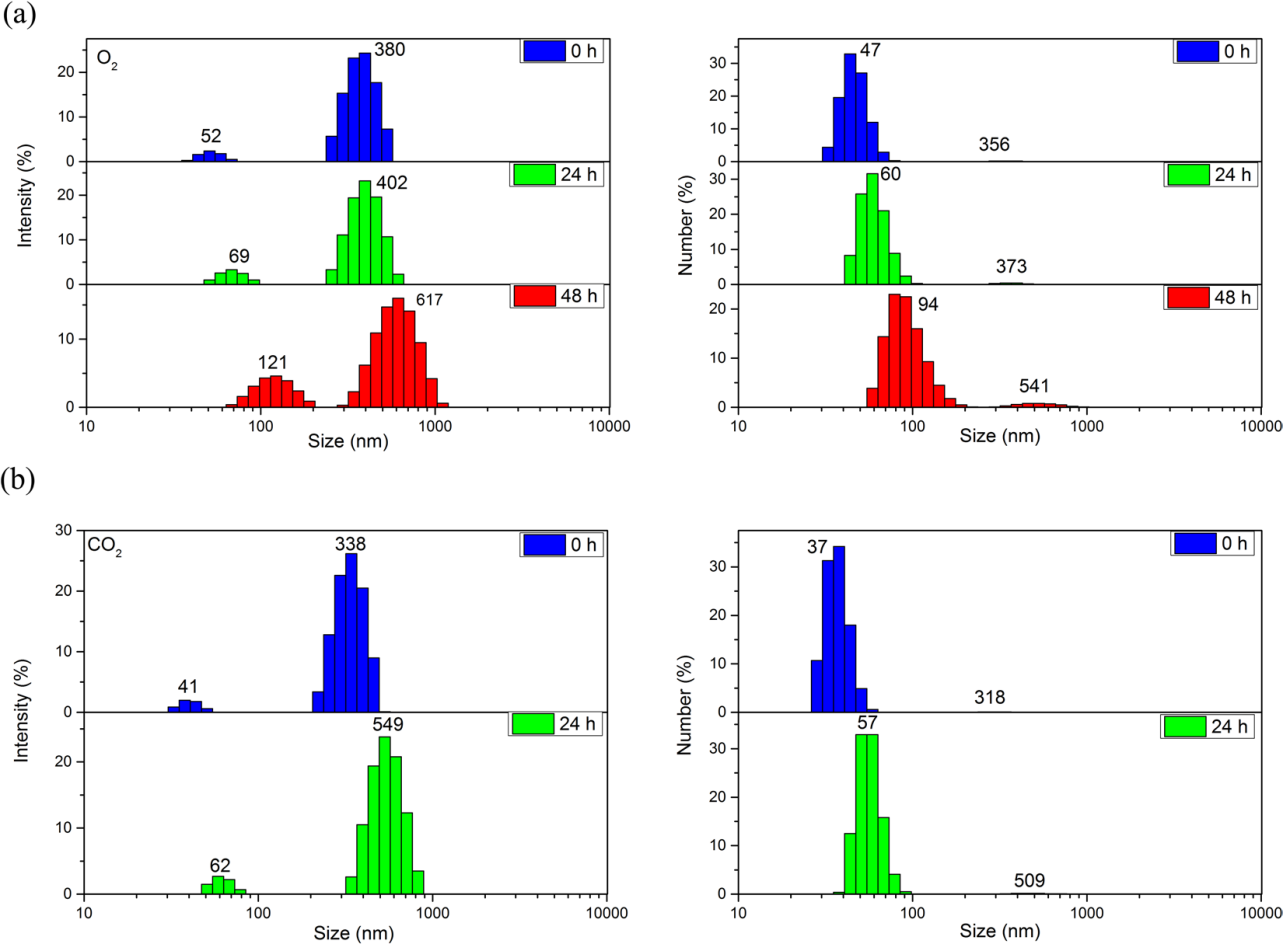
The size distributions of nanobubbles containing different gases with the elapsed time at 25 °C. (**a**) CO_2_ nanobubbles, (**b**) O_2_ nanobubbles.

The O_2_ and CO_2_ nanobubbles that may concern the various biological activities are also generated using this method, and their stability were investigated. Their size alterations over time were shown in Fig. 6. The two-peak profiles were displayed in size distribution, which looks similar to that of N_2_ nanobubbles, both have good stability and the size slowly grow as time. But compared with N_2_ and O_2_ nanobubbles, the lifetime of CO_2_ nanobubbles is less than 48 h observed from Fig. 6(b). Besides, it seem that the overall growth rates of O_2_ and CO_2_ nanobubbles were greater than that of N_2_ nanobubbles by comparing the change in size. The most probable diameters of O_2_ nanobubbles increased to 121 nm and 617 nm after 48 h, and CO_2_ nanobubbles were 62 nm and 549 nm after 24 h, although their initial mode diameter were slightly smaller than N_2_ nanobubbles after stopping generation.

According to the above results, the physicochemical properties of gas including the gas solubility, gas density and gas diffusion capacity, etc. may play an important role in managing the size of the formed BNBs^47,48^. Table S1 shows the related parameters about some properties of the gases used in this system. The density and solubility of carbon dioxide are higher than that of nitrogen and oxygen, especially solubility. While the size of the formed carbon dioxide BNBs is the smallest under the same condition, so the decision can be made that the nanobubbles formed from gas with high solubility may be smaller in this system. This phenomenon may provide insightful clue for understanding why and how the size of the formed BNBs could be adjusted.

### Discussion on the mechanism of bulk nanobubbles generation

As we all known, homogenous bubbles nucleation in bulk is difficult due to the high free-energy barrier. However, recently the existence of BNBs makes the situation different. To the best of our knowledge, supersaturation is significant for formation and stability of nanobubbles regardless of BNBs or SNBs^19,49^. In this study, the dispersion of nanobubbles was prepared on the basis of supersaturation realized through pressurization without high pressure equipment.

**Figure 7:**
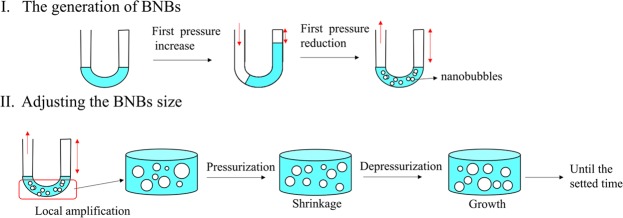
The diagram of possible mechanism of generating bulk nanobubbles by periodic pressure change at room temperature.

The detailed generation process of BNBs based on the periodic pressure change was discussed. Figure 7 shows the possible mechanism diagram of nanobubbles generation. The formation of nanobubbbles can be divided into two process: the generation and size adjustment. In first cycle of pressure change, when the syringe moved forward, the gas solubility in solution would increase with the increase of the pressure in U-shaped tube whose pressure eventually increase by around 0.8 atm. Then when the syringe moved backward, the bubble embryo is going to form and grow owing to the decrease in gas solubility in solution. Now the initial BNBs was generated. Because nanobubbles size that depend on the pressure reduce under pressurization^4,50,51^, in second cycle, the gas dissolution in nanobubbles tends to occur with the increase of pressure again, and the dissolved nanobubbles would grow again as the pressure decreases. This process of the periodic pressure change is similar to the response of surface nanobubbles to acoustic field^52^. In both cases, the gas would diffuse into bubble which is in expansion state caused by pressure reduction, while when bubble is in compression caused by pressure increase, the gas would diffuse out bubble going back to solution to be dissolved again. The shrinkage and growth of BNBs will continue until a given time is reached. Finally, the stable nanobubbles are formed.

By above discussion, the size of the formed BNBs would be affected by many factors, such as gas physicochemical properties, employing pressure, pressure change frequency, working time, etc. About how the gas solubility affect the size of the formed nanobubbles, the answer may be given here: In one respect, the gas solubility may affect the nucleation process of nanobubbles. Gas molecules with high solubility are closer to each other in solution, which makes the nanobubble nucleation easier to happen during decompression of first cycle, so smaller nanobubbles will be formed due to the more nucleation sites. In another respect, there is higher gas concentration gradient between nanobubbles and solution in the situation of high gas solubility, which may result in relatively high gas diffusion rate out from nanobubbles in the process of pressure increasing so that the formation of the smaller nanobubbles.

According to the understanding about the formation mechanism of the BNBs, it can be optimistically prospect that although there some complexity in controlling the size of nanobubbles in periodic pressure change, generating nanobubbles with appropriate size range may be realized by adopting the suitable action time of the periodic pressure change.

**Figure 8:**
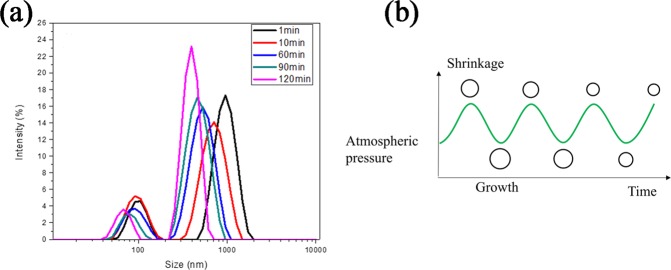
(**a**) The size distributions of N_2_ nanobubbles with various periodic pressure change time; (**b**) the diagram of change in BNBs size under pressurization and depressurization of the periodic pressure change.

To interpret the possibility of size-adjustable BNBs formation, the generation of N_2_ nanoububbles with various pressure change time were carried out. Figure 8 showed the variation of the nanoububbles size with different pressure change time. Unlike the growth behavior of SNBs in an acoustic pressure, the BNBs size gradually decreased with the increase of circulation time and the bubbles size reached its minimum when the time up to 120 min in our experimental time range. It may be attributed to the fact that the shrinkage rate under pressurization is greater than the growth rate under depressurization in a periodic pressure change, resulting in the shrinkage of BNBs over time. It demonstrates the size-adjustable nanobubbles can be achieved by managing the periodic pressure change time. The Fig. 8(b) showed the diagram of change in BNBs size under pressurization and depressurization respectively.

## Conclusions

In this study, bulk nanobubbles were successfully generated by employing periodic pressure change without high pressure equipment. The diameters of formed N_2_ nanobubbles were bimodal distribution with the peak values of around 68 nm and 397 nm. Furthermore, N_2_ nanobubbles have good stability with more than 48 h and the negative zeta potential experienced no significant change over 48 h. It was also found that the stability of N_2_ and O_2_ nanobubbles were better than CO_2_ nanobubbles. In addition, by manipulating pressure change time, the size of the formed nanobubbles could be adjusted. In summary, a convenient and reliable method of fabricating bulk nanobubbles was reported, which indicates that harsh conditions may not be required in BNBs formation. As a result, there may be a large quantity of nanobubbles existing in many practical productions, even in life activities, and may play a significant role. Therefore, it is meaningful to carry out the pertinent research on nanobubbles in these respects and perhaps some unknown important role can be uncovered in the future.

## Supplementary information

supplementary information

## Data Availability

All data generated or analyzed during this study are included in this published article and its supplementary information files.
