# Supplementary material for: Generation and Stability of Size-Adjustable Bulk Nanobubbles Based on Periodic Pressure Change
Source: Sci Rep. 2019 Feb 4;9:1118. doi: 10.1038/s41598-018-38066-5 (PMC6362149; doi:10.1038/s41598-018-38066-5)
Supplement: Supplementary file 1 — supplementary information [file 41598_2018_38066_MOESM1_ESM.docx]

**Supplementary Information**

**Generation and Stability of Size-Adjustable Bulk Nanobubbles Based on** **Periodic Pressure Change**

*Qiaozhi Wang*,^†^ *Hui Zhao*,^†^ *Na Qi*,^†^ *Yan Qin*,^†^ *Xuejie Zhang*^†^ and *Ying Li**^,†^

^†^Key Laboratory of Colloid and Interface Chemistry of State Education Ministry, Shandong University, 27 south of Shanda Road, 250100, Jinan, P. R. China

Contents

*Corresponding author

Tele: +86-531-88362078

Fax: +86-531-88364464

Email: [yingli@sdu.edu.cn](mailto:yingli@sdu.edu.cn)





Figure S1. The intensity distribution of ultrapure water without employing periodic pressure change.


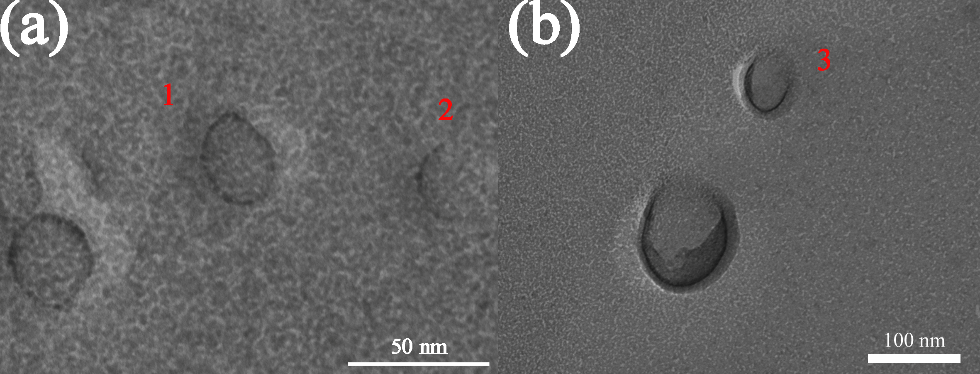


Figure S2. FF-TEM images of nitrogen nanobubbles formed in ultrapure water after employing the periodic pressure change for 120 min.

Table S1. The solubility in water and the density of nitrogen, oxygen and carbon dioxide at 25 ℃, 101.325 kPa^1^

| Gas | Solubility(10^5^ mol gas/mol H_2_O) | Density (g/L) |
| --- | --- | --- |
| N_2_ | 1.183 | 1.1449 |
| O_2_ | 2.293 | 1.3080 |
| CO_2_ | 61.5 | 1.7989 |

Reference

1. Lide, D. R. *RC Handbook of Chemistry and Physics, 84th ed; CRC Press:* Bocca Raton, Florida, 2003.
